# Carbamazepine Degradation Mediated by Light in the Presence of Humic Substances-Coated Magnetite Nanoparticles

**DOI:** 10.3390/nano9101379

**Published:** 2019-09-26

**Authors:** Francisca Aparicio, Juan Pablo Escalada, Eduardo De Gerónimo, Virginia C. Aparicio, Fernando S. García Einschlag, Giuliana Magnacca, Luciano Carlos, Daniel O. Mártire

**Affiliations:** 1 Instituto de Investigaciones Fisicoquímicas Teóricas y Aplicadas (INIFTA), Departamento de Química, Facultad de Ciencias Exactas, Universidad Nacional de la Plata, CONICET. Casilla de Correo 16, Sucursal 4, La Plata 1900, Argentina; faparicio@inifta.unlp.edu.ar (F.A.); fgeins@inifta.unlp.edu.ar (F.S.G.E.); 2 Unidad Académica Río Gallegos, Universidad Nacional de la Patagonia Austral, Río Gallegos 9400, Argentina; jescalada@uarg.unpa.edu.ar; 3 Instituto Nacional de Tecnología Agropecuaria (INTA), Estación Experimental Agropecuaria Balcarce, Route 226 Km 73,5, Balcarce 7620, Argentina; edg1981@gmail.com (E.D.G.); aparicio.virginia@inta.gob.ar (V.C.A.); 4 Dipartimento di Chimica and NIS Inter-departmental Centre, Università di Torino, Via Giuria 7, 10125 Torino, Italy; giuliana.magnacca@unito.it; 5 Instituto de Investigación y Desarrollo en Ingeniería de Procesos, Biotecnología y Energías Alternativas (PROBIEN), Neuquén 8300, Argentina

**Keywords:** magnetite nanoparticles, humic acids, carbamazepine, photo-Fenton, contaminants of emerging concern

## Abstract

The use of iron-based nanomaterials for environmental remediation processes has recently received considerable attention. Here, we employed core-shell magnetite-humic acids nanoparticles as a heterogeneous photosensitizer and iron source in photo-Fenton reaction for the degradation of the psychiatric drug carbamazepine (CBZ). CBZ showed low photodegradation rates in the presence of the magnetic nanoparticles, whereas the addition of hydrogen peroxide at pH = 3 to the system drastically increased the abatement of the contaminant. The measured Fe^2+^ and Fe^3+^ profiles point to the generation of Fe^3+^ at the surface of the nanoparticles, indicating a heterogeneous oxidation of the contaminant mediated by hydroxyl radicals. Products with a higher transformation degree were observed in the photo-Fenton procedure and support the attack of the HO^•^ radical on the CBZ molecule. Promising results encourage the use of the nanoparticles as efficient iron sources with enhanced magnet-sensitive properties, suitable for applications in photo-Fenton treatments for the purification of wastewater.

## 1. Introduction

As a consequence of the modern lifestyle, which includes the consumption of a wide variety of chemical products (pharmaceutical drugs, beauty products, recreational drugs, etc.), a new type of pollution emerged. This contamination is due to the so-called Contaminants of Emerging Concern (CECs), which are a group of chemical substances that have been recently detected in freshwaters. Perfluorinated compounds, pharmaceuticals, personal-care products, disinfection byproducts, sunscreens, paints, flame retardants, algal toxins, dioxane, pesticides and their degradation byproducts or nanomaterials are considered within this group [1,2,3]. The compound carbamazepine (CBZ, a psychiatric drug) is a CEC commonly found in natural waters, which is refractive to conventional water treatments and thus can be found in many rivers and streams [4,5,6]. For instance, CBZ was detected at concentrations ranging from ng L^−1^ to µg L^−1^ in samples of surface freshwaters taken from different rivers and lakes of Europe [7]. In Argentina, CBZ was found at concentrations in the range 0.2–2.3 µg L^−1^ in municipal wastewaters discharging into fresh and estuarine waters from areas with varying degrees of urbanization [8].

In general, CECs are hard to remove from waters and several research groups are thus developing different innovative methods to solve this issue. Among these, the advanced oxidation processes (AOPs) have been shown to be very efficient in the elimination of organic compounds refractory to biological treatments [9]. The AOPs are based on the generation of highly reactive species, such as hydroxyl radicals, which are able to react with the organic pollutant and degrade it to less toxic compounds [10]. In particular, the removal of CBZ has been widely investigated using different AOPs in recent literature [11,12].

Nanoparticles have shown promising performance in pollutant removal or toxicity mitigation. In particular, the use of iron-based nanomaterials for environmental remediation processes has recently received considerable attention [13,14,15,16]. The most widely used magnetic nanomaterials, magnetite (Fe_3_O_4_) and maghemite (γ-Fe_2_O_3_) nanoparticles, have gained great interest in research on engineering applications for the treatment of polluted water [17]. The use of magnetic nanoparticles in heterogeneous AOPs, such as photocatalysis and heterogeneous photo-Fenton processes, is gaining increasing interest since these materials aim to overcome the drawbacks of the separation and re-use of the catalyst [18,19]. We have shown that suspensions of humic acid (HA)-coated Fe_3_O_4_ nanoparticles are able to generate reactive oxygen species (ROS) upon irradiation with UV-visible light, and hence these materials are potential candidates for treating contaminants susceptible to HA-photosensitized degradation [20]. On the other hand, magnetite nanoparticles coated with humic acid-like biopolymers derived from composted urban biowaste were successfully employed as iron sources in the Fenton-like and photo-Fenton-like processes for the degradation of emerging contaminants at circumneutral pH [21,22]. However, the role of the organic coating of the nanoparticles in the photo-Fenton process is still not fully understood. 

The aim of the present work is to evaluate the ability of humic acid-coated magnetite nanoparticles as photosensitizers and also as iron sources in the photo-Fenton degradation of organic compounds. CBZ was used as model of CEC. Finally, photodegradation products were identified.

## 2. Materials and Methods 

### 2.1. Reagents

Carbamazepine (CBZ), FeCl_3_ (97%) and NH_4_OH (33%) were purchased from Sigma-Aldrich (Buenos Aires, Argentina). Ethanol (99.8%) and FeSO_4_·7H_2_O (99.5%) from Fluka (Buenos Aires, Argentina). o-phenanthroline (97%), KSCN (99%), 2-propanol (99.8%) and sodium acetate (99.9%) were from Anedra (Buenos Aires, Argentina) and H_2_O_2_ (30%) from Cicarelli (Buenos Aires, Argentina). Leonardite humic acid (LHA) was purchased from International Humic Substances Society (St. Paul, MN, USA). All chemicals were used without further purification. Water employed in all the experiments was ultrapure.

### 2.2. Synthesis and Characterization of Magnetic Nanoparticles

LHA-coated Fe_3_O_4_ nanoparticles (Fe_3_O_4_/LHA) were prepared according to the reported method [20]. Briefly, 4.17 g of FeSO_4_.7H_2_O and 6.22 g of FeCl_3_.6H_2_O were dissolved in 100 mL of deionized water and heated up to 90 °C. Then, 10 mL of 25% ammonium hydroxide and 50 mL of 1.0% *w*/*v* LHA were added. The mixture was stirred for 30 min and the temperature was kept at 90 °C. The black precipitate was purified using multiple water washes with magnetic separation from the supernatant. The bare Fe_3_O_4_ nanoparticles were prepared with a similar procedure except that no LHA was added.

Thermogravimetric curves were performed to estimate the organic matter content of the nanoparticles. A RIGAKU Thermo plus EVO instrument (Tokyo, Japan), under a nitrogen atmosphere with a heating rate of 10 °C min^−1^ from room temperature to 700 °C was used. Fourier transform infrared spectra (FT-IR) in the range from 4000 to 400 cm^−1^ were recorded using KBr pellets on a Nicolet 380 spectrometer (Thermo Electron Corporation, Madison, WI, USA). Spectra were obtained by accumulating 80 scans with a resolution of 4 cm^−1^. Transmission electron microscopy (TEM) images were obtained in a CM 200 microscope (Philips, Eindhoven, The Netherlands) equipped with an ultratwin objective lens and acceleration voltage of 200 kV. An *X*-ray diffraction (XRD) pattern was obtained by means of an X’Pert PRO MPD diffractometer from PANalytical (Malvern, UK), equipped with Cu anode, working at 45 kV and 40 mA, in a Bragg–Brentano geometry. The acquisition was performed in a 0.02° interval steps with 45 s step^−1^. UV-Vis absorption spectra of powder solid samples were recorded at room temperature with a double beam PG Instruments Limited T90+ spectrophotometer (Coventry, UK), equipped with an integrating sphere IS19-1. The solids were mixed with BaSO_4_ in a weight ratio of 1:5, the mixture was placed onto the sample holder and pressed to obtain a flat surface. Magnetization measurements were carried out with a LakeShore 7404 vibrating sample magnetometer (Westerville, Ohio, USA). The hysteresis loop of the sample was registered at 300 K and the magnetic field was cycled between −20,000 and 20,000 Oe. 

### 2.3. Kinetics of Carbamazepine (CBZ) Photodegradation

Photolysis experiments were carried out at room temperature under stirring in an RPR-100 Rayonet reactor (Bradford, CT, USA) equipped with eight RPR-3500 lamps. The spectral distribution of the lamps (Figure S1, Supporting Information) shows an emission centered at λ = 350 nm with a Full width at half maximum (FWHM) of 30 nm. 200 mL of the suspensions were irradiated within a 500 mL-cylindrical glass tube of 4 cm internal diameter. The incident photon rate measured using potassium ferrioxalate as actinometer [23], was 3.50 × 10^−5^ Einstein s^−1^ L^−1^. The concentrations of CBZ and Fe_3_O_4_/LHA varied from 2 to 20 mg L^−1^ and 300 to 1500 mg L^−1^, respectively. Experiments in the presence of H_2_O_2_ (0.15 mm) were also performed. During the experiments, samples were withdrawn at different irradiation times from the photochemical reactor and the supernatant obtained after magnetic separation was filtered with 0.45 μm nylon filters before quantification of CBZ and iron ions. The concentration profiles of CBZ were analyzed by HPLC (high performance liquid chromatography) using a Shimadzu instrument (Kyoto, Japan) equipped with a solvent delivery module LC-20AT, on-line degasser DGU-20A5, UV–vis photodiode array detector SPD-M20A, column oven CTO-10 A5 VP and autosampler SIL-20AAT. The column used was a Lichrospher RP-C18, 4 mm i.d. × 125 mm long (Hitachi Ltd., Tokyo, Japan). For the quantification of CBZ, the mobile phase was ACN-H_2_O 50/50 and the detection wavelength was 280 nm. The column temperature was maintained at 40 °C and a flow rate of 0.8 mL min^−1^ was used. Fe^2+^ and Fe^3+^ profiles were determined colorimetrically by using the reported methods of *o*-phenanthroline [24] and thiocyanate [25], respectively.

### 2.4. Reuse Test

Several cycles of Fe_3_O_4_/LHA reuse in the photo-Fenton treatment of CBZ degradation were performed. A typical procedure was as follows: a dispersion containing Fe_3_O_4_/LHA (500 mg L^−1^), CBZ (2 mg L^−1^) and H_2_O_2_ (0.15 mM) at pH 3.0 was irradiated for 10 min. After that, a magnetic separation step was performed in order to separate the solid from the supernatant. An aliquot was withdrawn for the determination of CBZ concentration and the remaining supernatant was discarded. Then, the second cycle consisted of the addition of CBZ and H_2_O_2_ solutions to the reactor containing the used nanoparticles, fixing the pH to 3.0 and irradiation of the mixture for 10 min. The same procedure was repeated several times. 

### 2.5. Product Analysis

An ultra-performance liquid chromatography-tandem mass spectrometer (UHPLC-MS/MS) analysis was performed using an ACQUITY UPLCTM system (Waters Corp., Milford, MA, USA) coupled to a Quattro Premier TM XE tandem quadrupole mass spectrometer (Waters, Manchester, UK). MassLynx 4.1 software equipped with QuanLynx software (Waters) was used to control the instruments and to process the data. An ACQUITY UPLC equipment consisting of a binary pump, an auto-sampler, and a column heater was used. Chromatographic separation was carried out on a UPLC BEH C18 (1.7 μm, 2.1 × 100 mm; Waters). Solvent A was water (0.1 mm NH_4_Ac/0.01% formic acid), and solvent B was methanol (0.1 mm NH4Ac/0.01% formic acid). The flow rate was set at 0.3 mL min^−1^ and the column temperature was 35 °C. An auto-sampler was used to inject 20 μL of the samples. The Quattro Premier TM XE tandem quadrupole mass spectrometer was operated in positive mode with the electrospray-ionization (ESI) source. The operating parameters were optimized under the following conditions: capillary voltage, 3.5 kV, ion source temperature, 120 °C, desolvation temperature, 450 °C, cone gas flow, 80 L h^−1^, desolvation gas flow, 800 L h^−1^ (both gases were nitrogen), and collision gas flow, 0.3 mL min^−1^ (argon gas). 

Multiple reaction monitoring (MRM) transitions, applied cone voltages, and collision energies are summarized in Table S1 of the Supporting Information. Liquid chromatography–mass spectrometry (LC-MS) full-scan was used to select a list of molecular ions whose levels are significantly altered between case and control samples. These molecules are then subjected to precursor-ion (PI) scans to acquire MS/MS data by manually setting the *m*/*z* values of the PIs.

## 3. Results

### 3.1. Characterization of Magnetic Nanoparticles

Figure S2, in the Supporting Information, shows TEM images of Fe_3_O_4_/LHA. It can be observed the occurrence of aggregates of roughly spherical crystalline nanoparticles of ca. 10 nm diameter surrounded by an amorphous layer of organic matter. XRD analysis was used to identify the iron oxides phases present in Fe_3_O_4_/LHA (Figure S3, in the Supporting Information). Diffraction peaks at 2θ = 30.1°, 35.4°, 43.0°, 53.9°, 57.2° and 62.6° are consistent with the magnetite pattern, corresponding respectively to the crystal planes (220), (311), (400), (422), (511), and (440) (card numbers 00-019-0629, ICCD Database). However, the presence of maghemite cannot be discarded since XRD is not an appropriate technique to discriminate between both phases because their XRD patterns are very similar.

The thermogravimetric analysis (TGA) obtained for Fe_3_O_4_/LHA nanoparticles (Figure S4 Supporting Information) shows a first weight loss (25–150 °C) due to evaporation of water, followed by the elimination of the chemically adsorbed organic coating in the 150–500 °C range. Data analysis shows that the amount of LHA sorbed on the surface of the Fe_3_O_4_ nanoparticles was 17.6%. The FT-IR spectrum of Fe_3_O_4_/LHA nanoparticles is shown in Figure S5 in the Supporting Information. The presence of magnetite was confirmed by a strong band at ca. 588 and 630 cm^−1^, which corresponds to the Fe–O stretching vibration [26]. The sharp signal of the C=O stretching at 1400 cm^−1^ is characteristic for carboxylate anions interacting with the FeO surface [27,28] and confirms the important role of these groups in the bonding of the LHA to the magnetite surface. These results are in agreement with those obtained for particles previously prepared in our laboratory under similar conditions [20].

The UV-visible absorption spectra of Fe_3_O_4_/LHA dispersed in water (2 g L^−1^) is shown in Figure S6, in the Supporting Information. The absorbance of Fe_3_O_4_/LHA is higher than that of bare Fe_3_O_4_ nanoparticles in the range 250–500 nm due to the presence of LHA on the surface of Fe_3_O_4_/LHA. The difference between both spectra increases with decreasing wavelength, in line with the reported spectra of humic substances [29].

Figure S7, in the Supporting Information, reports the magnetization curve obtained for Fe_3_O_4_/LHA at 300 K. The nanoparticles showed a superparamagnetic behavior with a magnetic saturation (Ms) of 55 emu g^−1^. This value is similar to those previously reported for magnetite nanoparticles coated with humic acid-like biopolymers derived from composted urban biowaste [28]. These magnetic properties indicate that the nanoparticles can be easily recovered by an external magnetic field.

### 3.2. Fe_3_O_4_/Leonardite Humic Acid (LHA) as Photosensitizers

**Figure 1:**
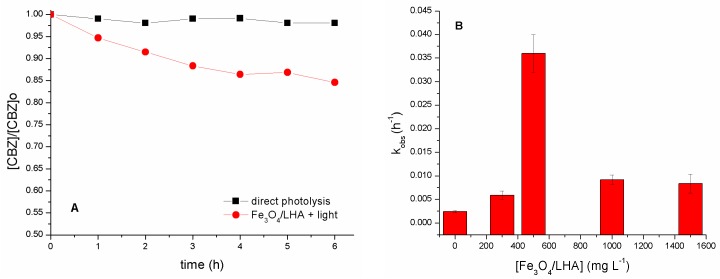
(**A**) Photodegradation of carbamazepine (CBZ) in the presence and in the absence of Fe_3_O_4_/Leonardite humic acid (LHA). (CBZ)_0_ = 2 mg L^−1^; (Fe_3_O_4_/LHA)_0_ = 500 mg L^−1^; pH 6.0. (**B**) Values of *k*_obs_ obtained for the photodegradation of CBZ solution for different initial concentrations of Fe_3_O_4_/LHA nanoparticles. (CBZ)_0_ = 2 mg L^−1^; pH 6.0.

The removal of CBZ after 6 h irradiation in the presence of 500 mg L^−1^ Fe_3_O_4_/LHA was 16% (Figure 1A). Direct photolysis and adsorption (dark experiments, not shown) do not significantly contribute to the removal of CBZ. These results clearly indicate the occurrence of indirect photoprocesses mediated by the Fe_3_O_4_/LHA nanoparticles and are in line with previously reported data obtained from irradiation of CBZ solutions in the presence of humic acids [30]. In order to evaluate the effect of the concentration of the nanoparticles, suspensions of Fe_3_O_4_/LHA in the range from 300 to 1500 mg L^−1^ containing 2 mg L^−1^ of CBZ were irradiated for 6 h. The degradation kinetic profiles were fitted to a pseudo-first order law. Although this simple model does not consider the complexity involved in this type of photoreactions, it can represent a useful tool for comparison purposes allowing the estimation of an observed rate constant (k_obs_) for each experimental condition. Figure 1B shows the values of k_obs_ obtained for different initial concentrations of Fe_3_O_4_/LHA. The highest CBZ removal (16%) and k_obs_ (0.036 h^−1^) was reached with the concentration of 500 mg L^−1^. Despite higher amounts of excited states of LHA and ROS are expected to be produced by increasing the concentration of suspended nanoparticles, for concentrations of Fe_3_O_4_/LHA higher than 500 mg L^−1^ the efficiency of CBZ degradation decreases. The latter result may be a consequence of the competition for the reactive species between CBZ and LHA bonded to the nanoparticles surface.

### 3.3. Fe_3_O_4_/LHA as Iron Sources in Photon-Fenton Treatment

**Figure 2:**
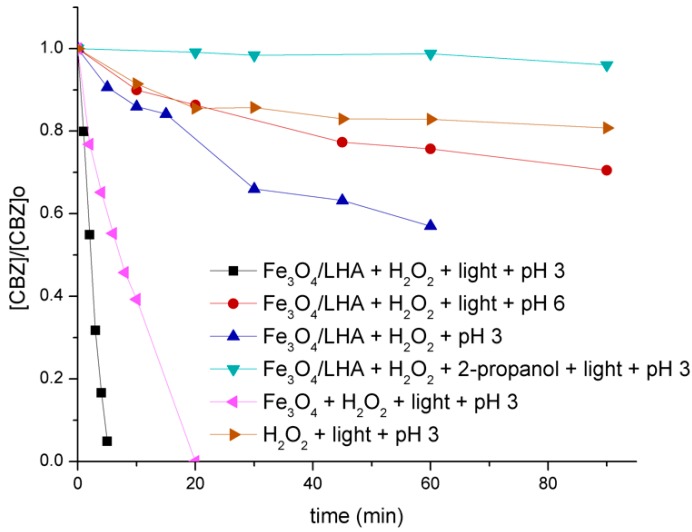
Normalized CBZ degradation under different experimental conditions. (CBZ)_0_ = 2 mg L^−1^; (Fe_3_O_4_/LHA)_0_ = 500 mg L^−1^, (Fe_3_O_4_)_0_ = 500 mg L^−1^, (H_2_O_2_)_0_ = 0.15 mM, (2-propanol)_0_ = 0.1 M when it is indicated.

To test the applicability of Fe_3_O_4_/LHA as an iron source in photo-Fenton experiments, 2 mg L^−1^ solutions of CBZ were irradiated in the presence of 500 mg L^−1^ Fe_3_O_4_/LHA and 0.15 mM H_2_O_2_ at pH 3.0 and 6.0. Only a CBZ degradation of ca. 25% was achieved after 60 min of irradiation at pH 6.0 (Figure 2). In contrast, under the same conditions but at pH 3.0, there was a complete depletion of CBZ within 5 min of irradiation (Figure 2). The degradation of CBZ was completely inhibited in the presence of 0.1 M 2-propanol, a well-known scavenger of hydroxyl radicals [31] (Figure 2). These results indicate a hydroxyl radical-mediated degradation of CBZ in these conditions. Experiments performed in the dark evidenced a small contribution of the Fenton reaction, because a 40% CBZ degradation was achieved after 1 h of reaction at pH 3 (Figure 2). This result shows that the iron species present in the Fe_3_O_4_/LHA nanoparticles can react with H_2_O_2_, but the UV light is necessary for an efficient degradation of CBZ in the presence of Fe_3_O_4_/LHA and H_2_O_2_. Control experiments performed by irradiating CBZ solutions in the presence of H_2_O_2_ at pH 3.0 (without Fe_3_O_4_/LHA added), indicate that the contribution of the HO^•^ radicals yielded by photolysis of H_2_O_2_ to the CBZ degradation in the photo-Fenton treatment is very low since only 20% CBZ removal was observed in this condition within the first 90 min of treatment (Figure 2). On the other hand, to ascertain the role of LHA on the surface of the nanoparticles, an experiment in the presence of bare Fe_3_O_4_ instead of Fe_3_O_4_/LHA was performed (Figure 2). Comparison between the experiments done with bare Fe_3_O_4_ and Fe_3_O_4_/LHA shows that although the humic acids on the surface of the nanoparticles can react with HO^•^ radicals [32], the lower CBZ degradation rate observed in the presence of bare Fe_3_O_4_ indicates that the presence of LHA enhances the CBZ degradation. This positive effect of LHA could be due to the following reasons: (i) stabilization of the magnetite nanoparticles avoiding their oxidation in water, and (ii) complexation of iron leached from the core of the nanoparticles during the UV irradiation, thus making the iron species more available to react with the H_2_O_2_ to yield HO^•^ radicals. 

**Figure 3:**
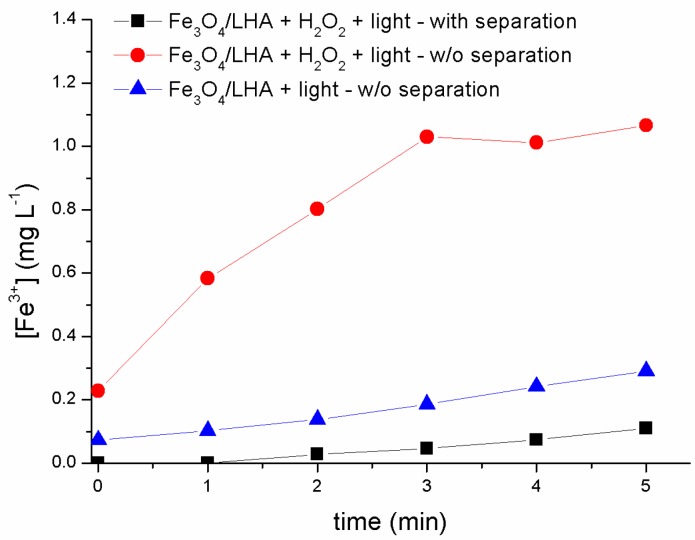
Fe^3+^ profiles measured in the absence and in the presence of H_2_O_2_, with and without the nanoparticle’s separation step. (CBZ)_0_ = 2 mg L^−1^; (Fe_3_O_4_/LHA)_0_ = 500 mg L^−1^; (H_2_O_2_)_0_ = 0.015 mM; pH 3.0.

The excellent degradation results of CBZ obtained when Fe_3_O_4_/LHA is used as an iron source in photo-Fenton experiments motivated us to investigate whether the generation of hydroxyl radicals takes place at the solvent-nanoparticle interface or in the bulk solution due to iron leaching from Fe_3_O_4_/LHA. Hence, the concentrations of Fe^2+^ and Fe^3+^ were measured at different reaction times in the supernatant after magnetic removal of the nanoparticles and filtration with a 0.45 µm Midland Scientific filter (Midland, TX, USA). The filtration step was performed in order to guarantee the assessment of soluble Fe^2+^ and Fe^3+^ species from the supernatant. For comparison purposes, Fe^2+^ and Fe^3+^ concentrations were also determined in the raw suspensions, i.e., without the magnetic separation process and filtration step. To this end, two series of experiments were performed in order to evaluate the evolution of the Fe^2+^ concentration upon irradiation of samples containing 0.15 mM H_2_O_2_ and 500 mg L^−1^ of Fe_3_O_4_/LHA at pH 3.0 in the absence of contaminants. We were unable to detect the generation of Fe^2+^ both in the filtered and unfiltered samples. This result suggests that either there is no Fe^2+^ leaching from the nanoparticles or it is consumed by the H_2_O_2_ present in the medium, leading to a Fe^2+^ concentration lower than the detection limit of the method (0.05 mg L^−1^). The Fe^3+^ profiles (Figure 3) resulting from the analysis of the filtered and unfiltered supernatant are quite different. The concentrations of Fe^3+^ measured in the filtered samples were one order of magnitude lower than those detected in the nanoparticles suspensions, which means that most of Fe^3+^ ions generated by the reaction between Fe^2+^ and H_2_O_2_ remain adsorbed on the nanoparticles. The low Fe^3+^ concentrations measured when the supernatant is not separated in experiments performed in the absence of H_2_O_2_ support the participation of the reaction between Fe^2+^ and H_2_O_2_ in our experimental conditions.

**Figure 4:**
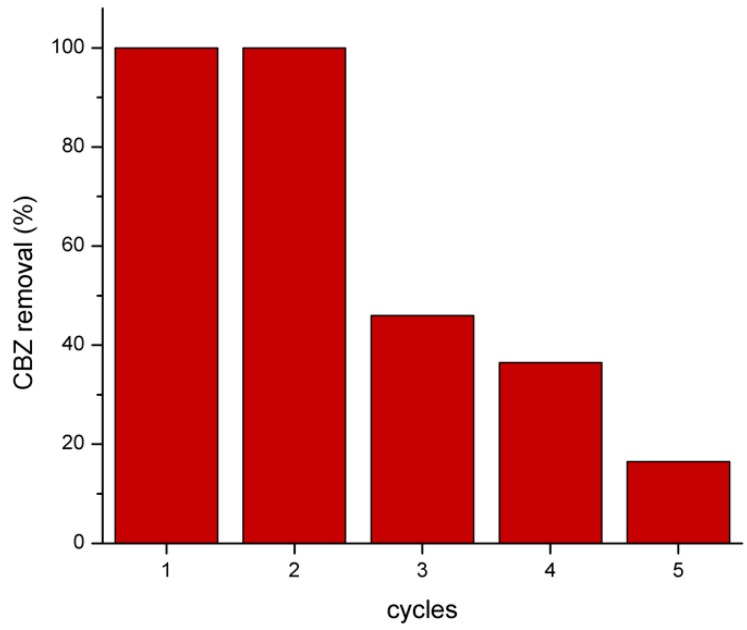
CBZ degradation in the presence of Fe_3_O_4_-LHA and H_2_O_2_ after several 10 min-cycles of UV irradiation. (CBZ)_0_ = 2 mg L^−1^; (Fe_3_O_4_/LHA)_0_ = 500 mg L^−1^; (H_2_O_2_)_0_ = 0.15 mM, pH = 3.0.

The reusability test of Fe_3_O_4_/LHA in photo-Fenton experiments was performed at pH 3 (Figure 4). The CBZ concentration was monitored after several 10 min-irradiation cycles. The nanoparticles retained their activity in the second cycle of reuse reaching a complete CBZ removal. However, after the third cycle, Fe_3_O_4_/LHA decrease their capacity and CBZ degradations lower than 50% are obtained. These results indicate that the Fe_3_O_4_/LHA have a limited reuse capacity probably due to iron leaching.

### 3.4. Product Analysis

**Figure 5:**
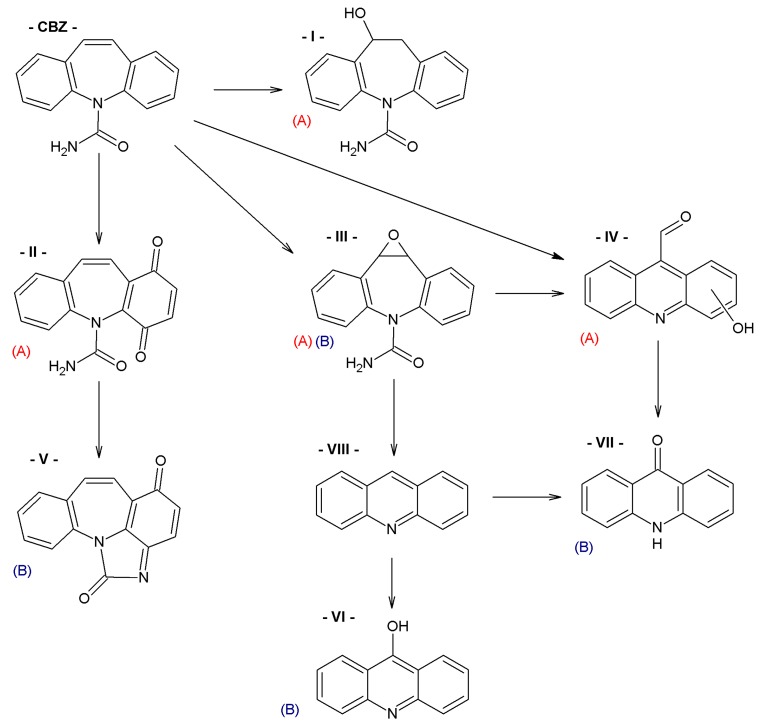
CBZ degradation pathways photo-induced by Fe_3_O_4_/LHA in the absence (A) and presence (B) of H_2_O_2_.

In order to obtain mechanistic information on the CBZ degradation under the different photochemical treatments tested, the reaction products were analyzed by UPLC-MS. Table 1 shows the CBZ photodegradation products obtained from assays performed both in the absence and presence of H_2_O_2_. On the basis of their chemical structures, Figure 5 shows possible CBZ degradation pathways associated with each process.

Compounds I–IV, listed in Table 1, were found for CBZ degradation induced by Fe_3_O_4_/LHA nanoparticles under UV irradiation. Product I was formed by the hydroxylation of the C_10_-C_11_ double bond of CBZ. This product was also detected as a result of the irradiation of CBZ both in the absence and in the presence of Fe^3+^ by other authors [33]. Thus, under our experimental conditions, this product could be a result of direct photolysis or it could be formed after reaction between CBZ with hydroxyl radicals. The quinonid (compound II) might form upon oxidation of an undetected hydroquinone structure, arising from two hydroxylation steps of the parent CBZ [33]. On the other hand, 10,11-epoxycarbamazepine (product III) is another observed intermediate derived from the reaction between hydroxyl radical and CBZ [34]. Formation of product IV involves a ring contraction process followed by a hydroxylation step. According to Chiron et al. [33] this product could arise from carbamazepine photoionization followed by hydroxylation at the 10 position or by the direct reaction between CBZ and HO^•^ radical, and further contraction of the 7-atom ring followed by a hydroxylation step, accompanied by loss of the -CONH_2_ lateral chain (see Figure S8 in the Supporting Information). Also, the subsequent opening of the epoxide ring of product III would give a labile species that undergoes facile ring contraction to finally yield product IV [34].

In the presence of Fe_3_O_4_/LHA and H_2_O_2_ under UV irradiation at pH 3.0, four degradation products were detected, and their structures were assigned (compounds III, V, VI and VII, Table 1). Compound V is a byproduct with a high degree of transformation. Losses of 28 mass units, corresponding to CO, are characteristic of quinone derivatives, suggesting that product V can be formed from compound II. An intramolecular reaction with H_2_O loss might then be responsible for the transformation of II into V. This product was also observed by irradiation of CBZ in the presence of Fe^3+^ at pH 2 by Chiron et al. [33]. The MS spectrum of the product with (M + H)^+^ at *m/z* = 196 shows fragments at *m/z* 167 and 168. This fragmentation is compatible with either 9-hydroxyacridine (compound VI) or acridone (compound VII) [35]. 9-hydroxyacridine is a typical hydroxylation product of acridine (compound VIII, Figure 5). Although acridine was not detected, its formation as intermediate during either photodegradation or oxidative treatments of CBZ is well-documented by other authors [33,34,35,36,37]. Acridone (VII) could also be formed by further oxidation of product IV through elimination of a carbonyl group [38]. Formation of acridone (VII) was also observed upon the attack of CBZ by HO^•^ radicals formed either by UV/H_2_O_2_ or UV/Fe^2+^ treatment [39].

It is noteworthy that different products of CBZ oxidation are obtained when Fe_3_O_4_/LHA are used as photosensitizers or as catalysts for photo-Fenton treatment. Products with higher transformation degree were observed in the photo-Fenton procedure, which is logical due to the higher observed degradation yields of CBZ. The only common product is compound III, which supports the attack of the HO^•^ radical on the CBZ molecule. This is reasonable because the hydroxyl radical besides being the main reactive species involved in the photo-Fenton treatment, is also generated upon photoirradiation of suspensions of the Fe_3_O_4_/LHA [23].

## 4. Conclusions

The core-shell magnetite-humic acids nanoparticles have been used on the one hand as heterogeneous photosensitizers and on the other hand as catalysts in the photo-Fenton treatment for the oxidation of carbamazepine. Carbamazepine showed a 15% photodegradation with UVA light in the presence of the magnetic nanoparticles and control experiments indicate that the contribution of direct photolysis was negligible under these conditions. The nanoparticles were proved to be excellent iron sources to be employed for the photo-Fenton degradation of organic contaminants. The Fe^2+^ and Fe^3+^ profiles measured in the filtered and unfiltered suspensions are indicative of a heterogeneous hydroxyl radical generation in the photo-Fenton process, which is responsible for the oxidation of the contaminants. Products with higher transformation degree were observed in the photo-Fenton procedure and the only common product obtained under both treatments is 10,11-epoxycarbamazepine, compound number III, which supports the attack of the HO^•^ radical on the CBZ molecule.

## Figures and Tables

**Table 1:** CBZ photodegradation products detected by UPLC-MS-MS.

Product Number	Formula	Molecular Ions (M + H)^+^, (*m/z*)	Specific Fragments (*m*/*z*)	Type of Treatment	
				A	B
I	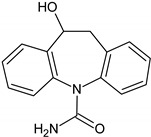	255	237	√	
II	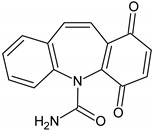	267	249	√	
III	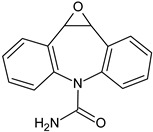	253	210, 180	√	√
IV	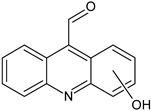	224	196	√	
V	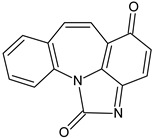	251	223, 208, 180		√
VI	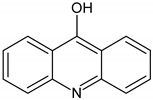	196	167, 168		√
VII	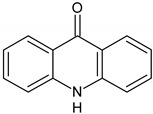	196	167, 168		√

(A) CBZ solution (2 mg L^−1^) irradiated 24 h in the presence of Fe_3_O_4_-LHA (500 mg L^−1^) at pH 3.0. (B) CBZ solution (2 mg L^−1^) irradiated 30 min in the presence of Fe_3_O_4_-LHA (500 mg L^−1^) and H_2_O_2_ (0.15 mm) at pH 3.0.

## Supplementary Materials

The Supplementary Materials are available online at https://www.mdpi.com/2079-4991/9/10/1379/s1.
