# Supplementary material for: Carbamazepine Degradation Mediated by Light in the Presence of Humic Substances-Coated Magnetite Nanoparticles
Source: Nanomaterials (Basel). 2019 Sep 26;9(10):1379. doi: 10.3390/nano9101379 (PMC6836229; doi:10.3390/nano9101379)
Supplement: Supplementary file 1 [file nanomaterials-09-01379-s001.pdf]

SUPPORTING INFORMATION

Carbamazepine degradation mediated by light in the presence of humic substances-coated magnetite nanoparticles.

Francisca Aparicio, Juan Pablo Escalada, Eduardo De Gerónimo, Virginia C. Aparicio, Fernando S. García Einschlag, Giuliana Magnacca, Luciano Carlos, and Daniel O. Mártire

Number of pages: 7

Number of Figures: 7

Number of Tables: 1

Contents:

Emission spectrum of the lamps (Figure S1) ……………..………………………..…….………….S2

TEM images (Figure S2) ……………………………………………………..……………………..S3

XRD pattern (Figure S3) ……………………………………………………..……………………..S4

TGA curve (Figure S4) ………………………………………………………….……….............…S4

FT-IR spectrum (Figure S5) ……………………………………………..….….……………………S5

UV-Vis absorption spectra (Figure S6) ……………………………………..….….…………………..S6

Magnetization curve (Figure S7) ………………………………..……….….….…………………..S6

Reaction mechanism (Figure S8)…………………………………………………...…………………S7

UPLC-MS/MS conditions for CBZ (Table S1)…………………………………………………………..S7

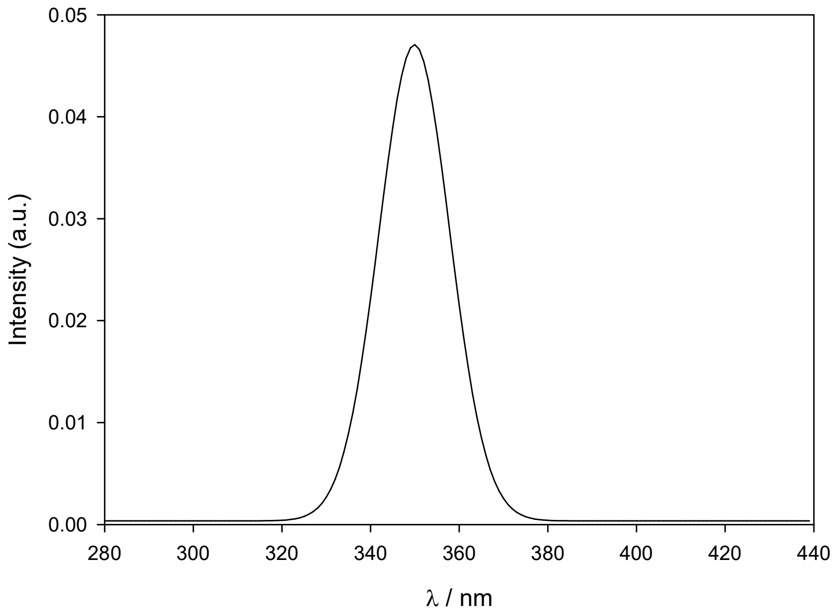

Figure S1. Emission spectrum of the lamps.

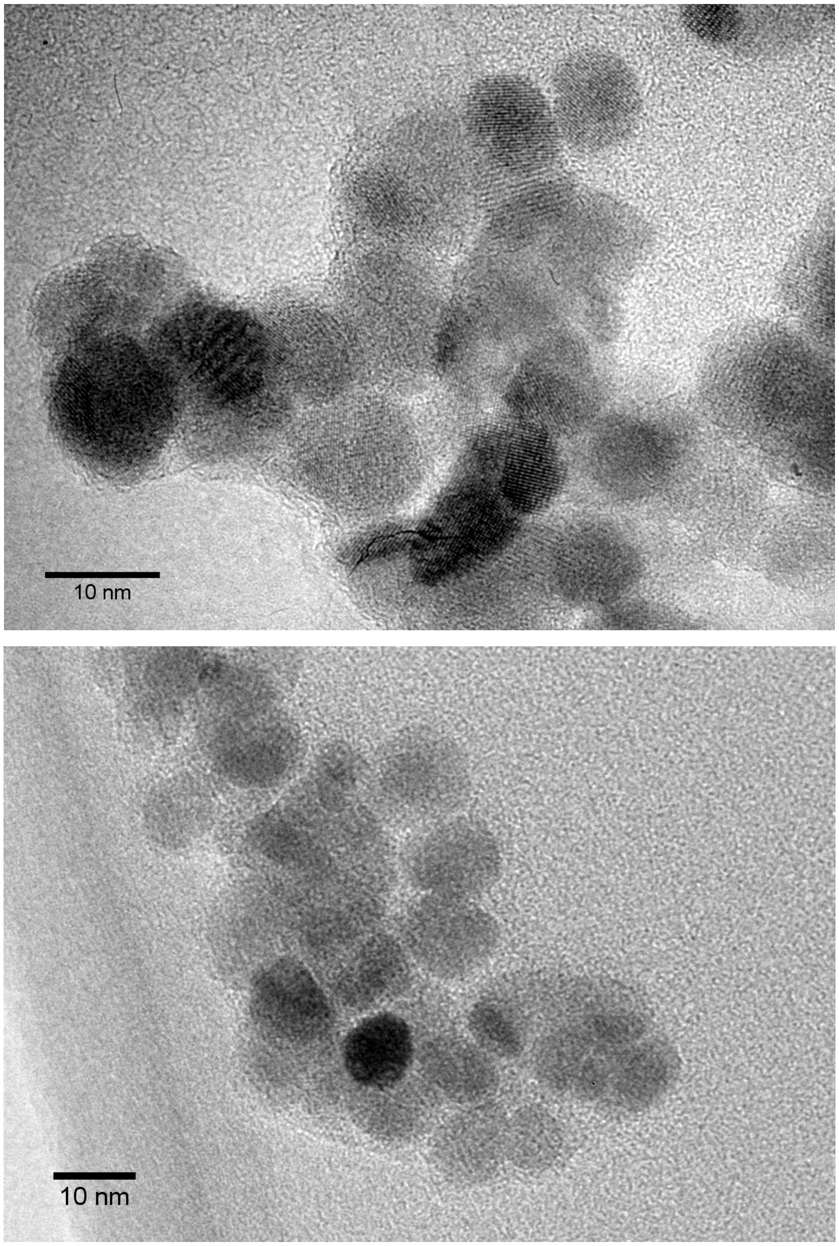

Figure S2. TEM images of Fe_3O_4/LHA.

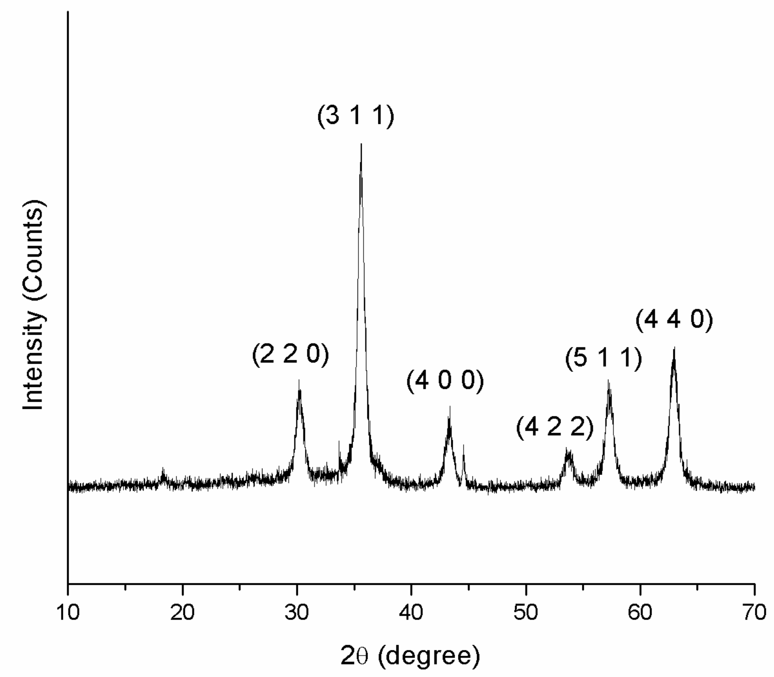

Figure S3. XRD pattern of Fe_3O_4/LHA. Magnetite/maghemite crystalline planes reflections are highlighted.

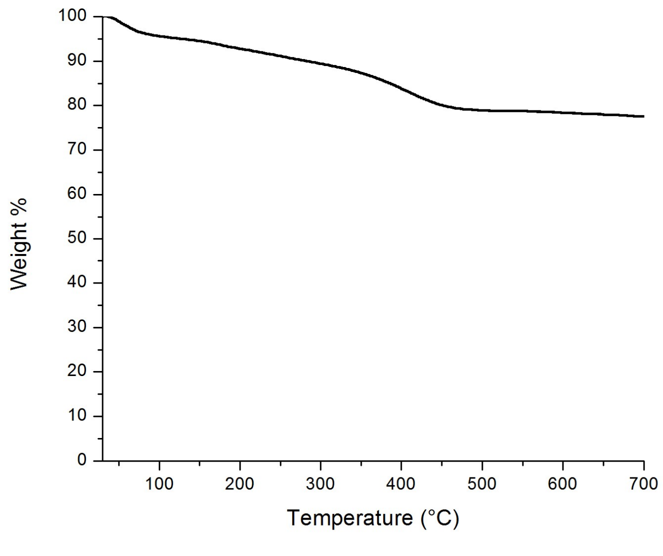

Figure S4. TGA curve obtained for Fe_3O_4/LHA under N_2 atmosphere.

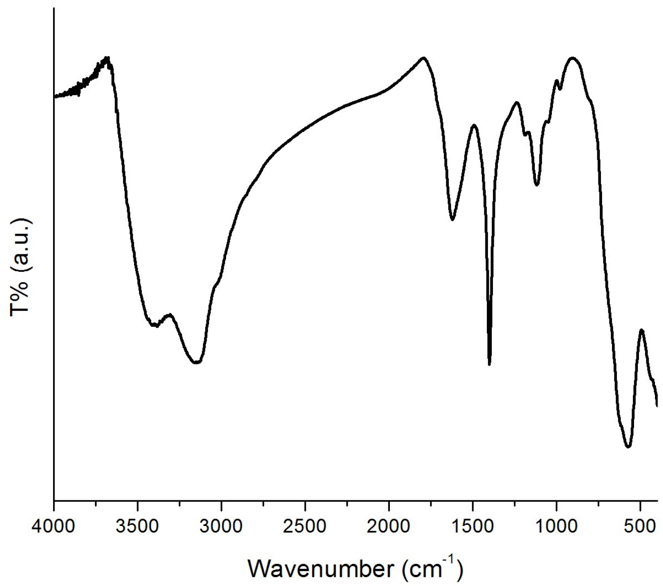

Figure S5. FT-IR spectra of Fe_3O_4/LHA nanoparticles.

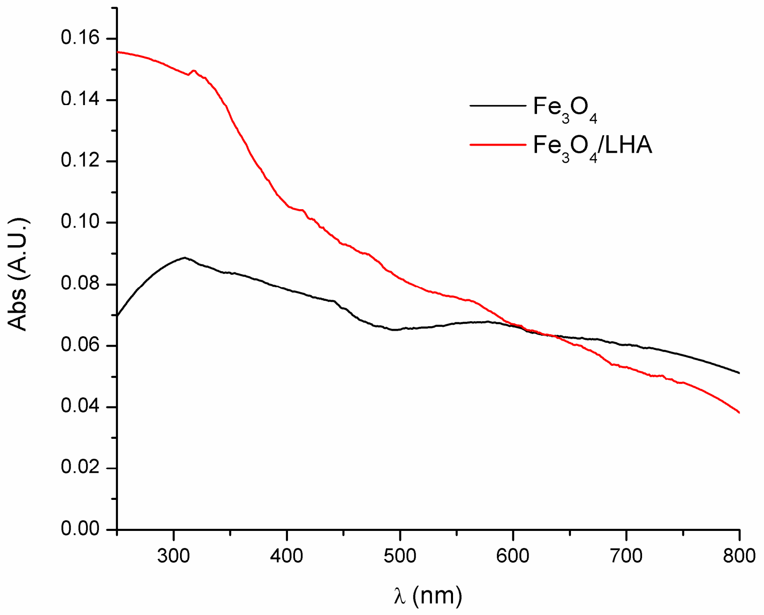

Figure S6. UV-Vis absorption spectra of Fe_3O_4 and Fe_3O_4/LHA.

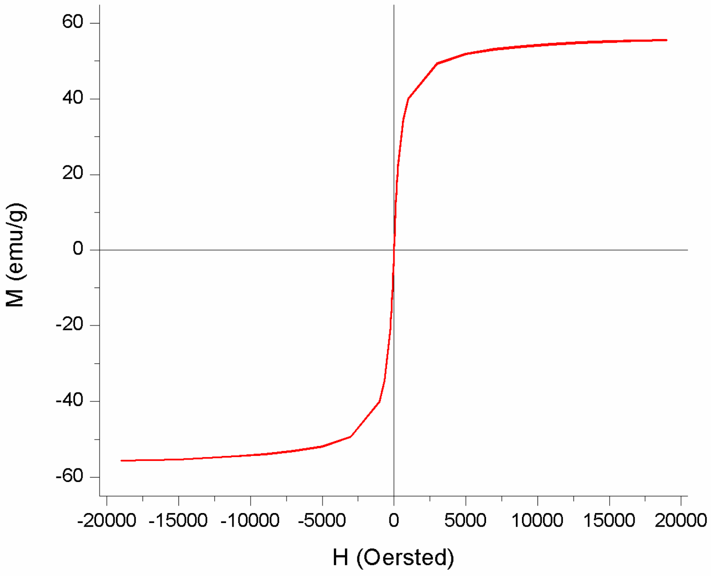

Figure S7. Magnetization curve of Fe_3O_4/LHA.

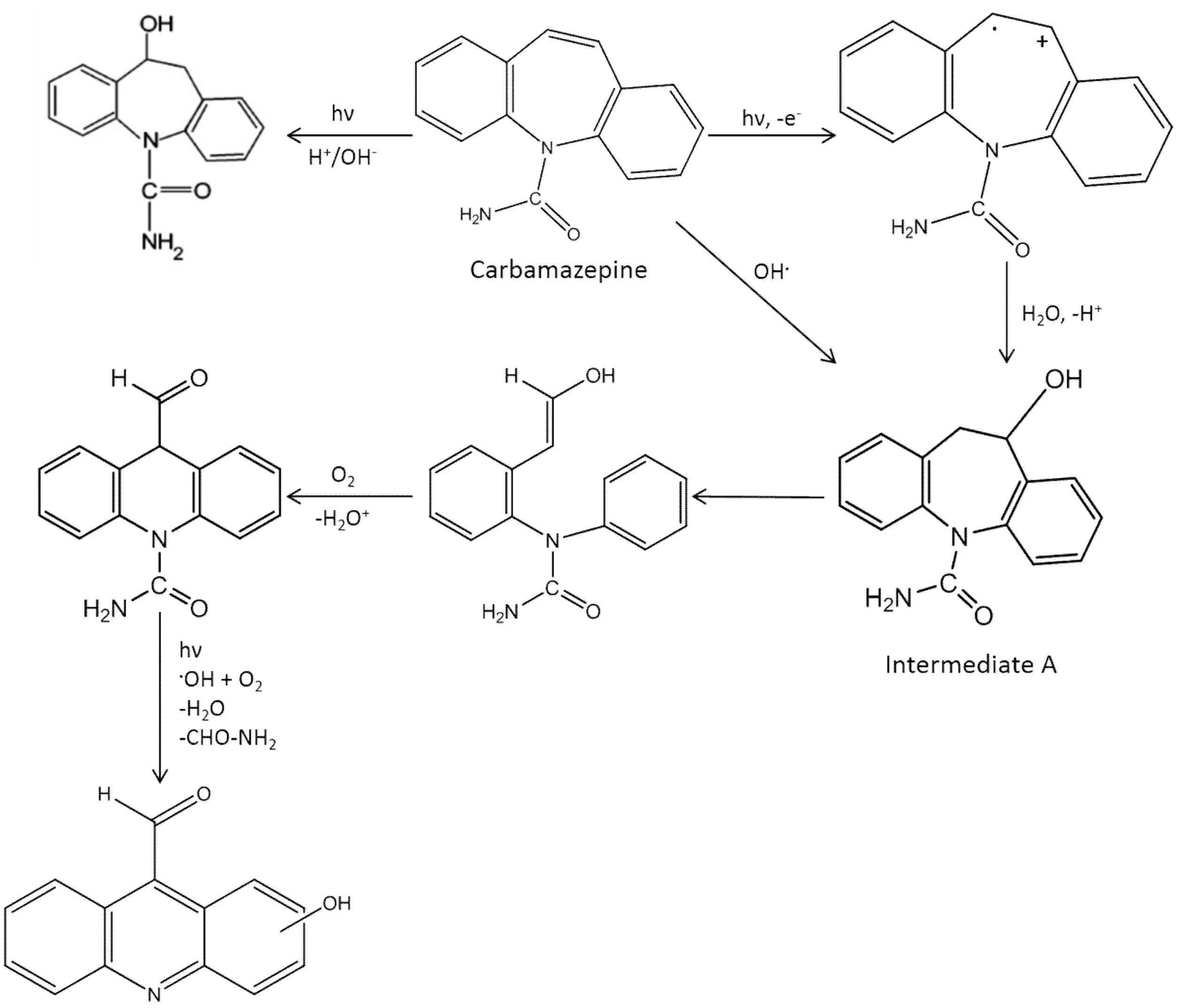

Figure S8: Reaction mechanism proposed for the formation of compound number IV.

Table S1. UPLC-MS/MS conditions for CBZ.

Compound	Cone voltage (V)	Precursor ion (m/z)	Quantification ion		Identification ion	
			Collision energy (eV)	Product ion (m/z)	Collision energy (eV)	Product ion (m/z)
Carbamazepine	26	236.8	20	191.9	20	193.9
